# Influences on participant reporting in the World Health Organisation drugs exposure pregnancy registry; a qualitative study

**DOI:** 10.1186/s12913-014-0525-1

**Published:** 2014-10-31

**Authors:** Elizabeth N Allen, Melba Gomes, Lucy Yevoo, Omar Egesah, Christine Clerk, Josaphat Byamugisha, Anthony Mbonye, Edwin Were, Ushma Mehta, Lynn M Atuyambe

**Affiliations:** Division of Clinical Pharmacology, Department of Medicine, University of Cape Town, Cape Town, South Africa; World Health Organisation, 1211 Avenue Appia, Geneva, 27 Switzerland; Dodowa Health Research Centre, Dodowa, Ghana; Department of Anthropology, Moi University, Eldoret, Kenya; School of Public Health, University of Ghana, Dodowa, Ghana; Department of Obstetrics and Gynaecology, Makerere University, Kampala, Uganda; Ministry of Health, Kampala, Uganda; Department of Reproductive Health, Moi University, Eldoret, Kenya; Independent Pharmacovigilance Consultant, Cape Town, South Africa; Department of Community Health and Behavioural Sciences, Makerere University School of Public Health, Kampala, Uganda

**Keywords:** Pregnancy, Registry, Drug safety, Ghana, Kenya, Uganda, Validity, Qualitative, Alcohol, Medicine use

## Abstract

**Background:**

The World Health Organisation has designed a pregnancy registry to investigate the effect of maternal drug use on pregnancy outcomes in resource-limited settings. In this sentinel surveillance system, detailed health and drug use data are prospectively collected from the first antenatal clinic visit until delivery. Over and above other clinical records, the registry relies on accurate participant reports about the drugs they use. Qualitative methods were incorporated into a pilot registry study during 2010 and 2011 to examine barriers to women reporting these drugs and other exposures at antenatal clinics, and how they might be overcome.

**Methods:**

Twenty-seven focus group discussions were conducted in Ghana, Kenya and Uganda with a total of 208 women either enrolled in the registry or from its source communities. A question guide was designed to uncover the types of exposure data under- or inaccurately reported at antenatal clinics, the underlying reasons, and how women prefer to be asked questions. Transcripts were analysed thematically.

**Results:**

Women said it was important for them to report everything they had used during pregnancy. However, they expressed reservations about revealing their consumption of traditional, over-the-counter medicines and alcohol to antenatal staff because of anticipated negative reactions. Some enrolled participants' improved relationship with registry staff facilitated information sharing and the registry tools helped overcome problems with recall and naming of medicines. Decisions about where women sought care, which influenced medicines used and antenatal clinic attendance, were influenced by pressure within and outside of the formal healthcare system to conform to conflicting behaviours. Conversations also reflected women's responsibilities for producing a healthy baby.

**Conclusions:**

Women in this study commonly take traditional medicines in pregnancy, and to a lesser extent over-the-counter medicines and alcohol. The World Health Organisation pregnancy registry shows potential to enhance their reporting of these substances at the antenatal clinic. However, more work is needed to find optimal techniques for eliciting accurate reports, especially where the detail of constituents may never be known. It will also be important to find ways of sustaining such drug exposure surveillance systems in busy antenatal clinics.

## Background

Pregnancy exposure registries are observational studies measuring the association between drugs taken during pregnancy and the pregnancy outcome [1]. They make important contributions to evidence on the safety of medicines in pregnancy, particularly as this vulnerable population is rarely included in clinical trials [2]. There is a specific need to establish the safety profile of life-saving therapies in countries with a high burden of diseases such as malaria, TB and HIV/AIDS, so that pregnant women are neither denied access to a safe drug nor exposed to an unsafe drug. For instance, with the recent rapid scale up in access to artemisinin-based combination therapies (ACT), effective pharmacovigilance during pregnancy is important as these drugs are contraindicated in the first trimester [3,4]. There is also much to be learnt about the safety of non-prescription medicines, including herbs, which are widely used but rarely studied [5-7].

**Figure 1:**
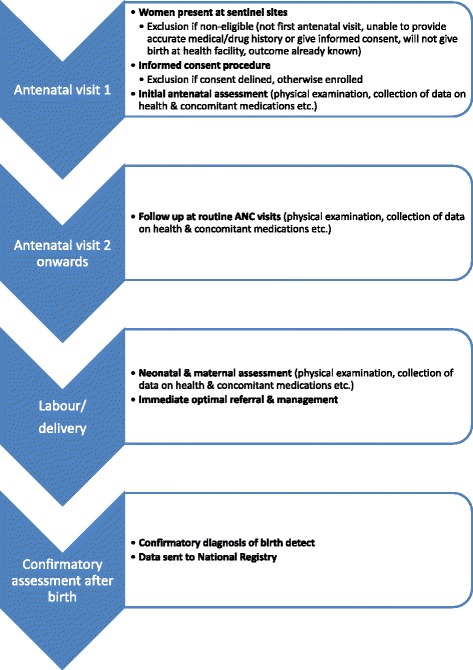
**Flow diagram of WHO pregnancy registry procedures.**

Data about the prevalence of birth defects, including those related to substances used post-conception, are generally lacking in resource-limited settings (middle and low income countries) so the World Health Organisation (WHO) designed a registry suitable for implementation by national health authorities in these contexts for routine surveillance [8,9]. The WHO registry, which is neither disease nor drug-specific, involves the prospective collection of detailed health and exposure data from a woman's first antenatal clinic (ANC) visit until delivery (Figure 1). As data accumulate within countries or regions, it is expected that the risk of specific drug exposures as determinants of maternal and neonatal outcomes may be quantified. In addition, the registry is intended to help build capacity for improved maternal and neonatal care within the health care systems. This could include training ANC staff about the importance of obtaining a good treatment history from pregnant women, collecting various indicators of maternal and new-born health, and strengthening referral systems for babies born with poor health.

As with all pharmacoepidemiologic studies the success of the WHO registry relies, largely, on the accuracy of exposure, outcome and covariate data [10]. In countries where there is a relatively poor infrastructure for maintaining institutional medical, prescription or dispensing records, it is difficult to derive objective measures of medical history and drug use [11]. Maternal reports may be the only source of these data, particularly for medicines obtained from private clinics, shops and traditional practitioners. Methodological research shows that demographic characteristics (such as age and socio-economic indicators), recall abilities, perceptions about the significance or relevance of the information, anticipation of consequences, and the ability to articulate a response, are potential factors involved in shaping patients' or research participants' reporting of health problems and medicine use [10,12,13]. The way questions are posed can also influence responses. For instance questionnaires that include indication-for use and drug-specific questions increase prevalence estimates for drug use compared to open-ended questions [14]. As barriers to accurate and complete reporting are likely to be context-specific it is important to investigate these in the area where research is conducted, such that relevant measures may be taken to improve data quality.

A qualitative component was included in the WHO registry pilot study in Ghana, Kenya and Uganda to examine the factors that either enabled or hindered the process of creating and maintaining a successful registry in low and middle-income settings. Specifically, it sought to explore, from pregnant women's perspectives, barriers to their reporting, and thereby staff recording, of drug use data in the registry and how these might be overcome; what types of information are under- or inaccurately reported at ANC visits, the underlying reasons, and how women would prefer to be asked questions. This paper describes the results of these research questions to inform optimal implementation of pregnancy registries by health authorities in similar contexts.

## Methods

We employed a qualitative study design, using focus group discussions (FGDs) to collect the data. These were nested within a WHO registry pilot study and conducted between July 2010 and August 2011. In each registry site a sample of women attending their first ANC visit were enrolled. ANC nurses in the registry teams were given standardised training in how to elicit information on medical histories and medicine use from enrolled women using a data collection tool which complemented the existing ANC records [8]. A similar tool, with additional fields related to the birth outcome, was used at the time of delivery. Women were also encouraged to maintain a written record of the names of medicines they had used (in notebooks), or collect samples or packets in plastic envelopes supplied. These were then to be brought to the site at routine ANC follow-up visits to help registry staff make an accurate record.

### Qualitative study areas, population and sampling

The study population for this qualitative component included two main categories of women 1) women of all ages who had been enrolled in the WHO pregnancy registry pilot study being conducted at selected sites in Ghana (Dangme West District Hospital), Kenya (Webuye District Hospital) and Uganda (Iganga District Hospital) and 2) women of child-bearing age, who had experienced at least one pregnancy, living in the communities or catchment areas from which participants for the registry were drawn. The latter category was included to potentially detect influence of the registry methods on reporting. Each site was situated in a rural or semi-rural (small town) location. National figures for women's use of an ANC for at least 4 visits during pregnancy were 78% in Ghana (2008), 47% in Kenya (2009) and 48% in Uganda (2011); 55% (2008), 44% (2009) and 58% (2011) of women respectively are attended to by a skilled birth attendant during delivery; for 2011 the prevalence of HIV (age 15–49) and annual number of malaria cases are 1.5% and 1,041,260 for Ghana, 6.2 and 1002805 for Kenya and 7.2% and 231,873 for Uganda respectively [15].

There were 12 strata of FGDs, as participants were stratified by country, whether enrolled in the registry or from the registry's source community, and < or ≥24 years old (the latter because younger women often feel less comfortable in expressing themselves in the presence of older women [16]). Sampling was purposive as regards the strata and where community participants resided in relation to the respective ANCs. Those enrolled in the registry were accessed through liaison with the pilot registry ANC study staff teams, while women in the source community groups were recruited within the catchment areas of each antenatal clinic through households and community groups such as markets. Two to 3 FGDs were to be conducted per strata, with a minimum of 6 participants in each.

### Study conduct, data management and analysis

Senior local social scientists (LY, OE, LMA) supervised the field conduct of FGDs by teams trained according to a manual and standard operating procedures developed by an international coordinator (ENA). The original English version of the FGD question guide was translated and pre-tested in at least one FGD per site to ensure the terminology was locally appropriate. Topics included experiences and perceptions about treatment-seeking in pregnancy, poor birth outcomes, maintaining health and treatment records, the pregnancy registry and reporting information at the ANC. As recall ability is related to the time since exposure to a medicine or other substance it was also important to understand the influences on seeking adequate antenatal care, particularly the first ANC visit, and the types of medicines used. The FGDs were held close to the ANC but in a private space. Audio recordings of each FGD were transcribed directly into English using a meaning-based method, checked for quality and imported into NVivo 9 (QSR International Pty Ltd, 2011). ENA and each country social scientist co-coded at least 2 transcripts (deductively, using the research questions, and inductively) to agree on an initial coding framework before ENA completed coding for all countries. Each transcript was read several times before relevant text was examined for repeating concepts (codes) which were labelled and grouped into categories and themes reflecting the underlying meaning behind statements [17]. Quotes that represented the categories and themes were then selected for inclusion in this manuscript. Code counts were also used to express the size of some categories.

### Ethical considerations

Approval from the ethical committees or boards of the following institutions was obtained before the WHO pilot registry study started in each respective country: the WHO, Ghana Health Service, Moi Teaching and Referral Hospital, Makerere University Faculty of Medicine, and the Uganda National Council for Science and Technology. The University of Cape Town Human Research Ethics Committee also approved the analysis plan. Informed consent processes and forms for the FGDs were available in the local languages and, though information could be explained in a group, each woman met with a facilitator to confirm consent. Participants were informed about who would have access to the data collected, that refusal to participate or withdrawal of consent wouldn't affect their medical care, and that they did not need to discuss anything they were not willing to. No participant withdrew consent. Refreshments were provided during FGDs.

## Results

**Figure 2:**
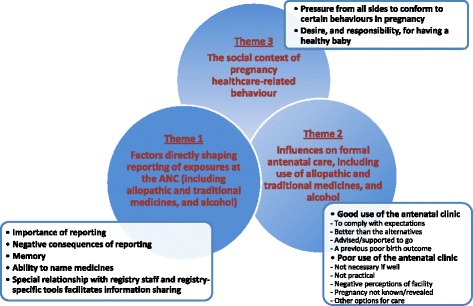
**Summary of the influences on participant reporting in the World Health Organisation drugs exposure pregnancy registry.**

Twenty-seven FGDs were conducted across the 3 study sites with a total of 208 women (Table 1). Ghana was unable to achieve 2 FGDs for registry participants and compensated by enrolling more women in the community FGDs. The qualitative analysis revealed three themes: "factors directly shaping reporting of exposures at the ANC (allopathic and traditional medicines, and alcohol) "," influences on formal antenatal care" and "the social context of pregnancy healthcare-related behaviour". Categories within these themes included: "importance of reporting", "negative consequences of reporting", "memory", "ability to name medicines", "special relationship with registry staff and registry-specific tools facilitates information sharing; "good use of the ANC", "poor use of the ANC" (with their codes); "pressure from all sides to conform to certain behaviours in pregnancy and "desire, and responsibility, for having a healthy baby". These are presented diagrammatically in Figure 2, while differences between the strata are described below.

**Table 1:** **Focus group discussion (FGD) participants in each strata**

	**Ghana**				**Kenya**				**Uganda**			
	**Registry**		**Community**		**Registry**		**Community**		**Registry**		**Community**	
**Age strata (years)**	<24	≥24	<24	≥24	<24	≥24	<24	≥24	<24	≥24	<24	≥24
**Number of FGDs**	1	1	2	4	2	2	2	2	2	3	3	3
**Number of participants per strata**	9	9	16	40	13	12	20	21	11	19	19	19
**Mean age (range) in years**	21 (18–23)	31 (26–41)	21 (18–23)	38 (24–72)	20 (NK)	30 (NK)	23 (NK)	30 (NK)	21 (18–23)	27 (24–35)	21 (17–23)	31 (25–40)
**Mean number of children**	0	2	1	4	0	2	1	3	1	2	1	3

NK = not known.

### Factors directly shaping reporting of exposures at the ANC (allopathic and traditional medicines, and alcohol)

Two large categories within this theme (across all countries, ages and whether in the community or registry groups) were conflicting; women said that it was important to report everything relating to their health at the ANC for their own and their baby's benefit (31% of relevant codes). However, this could have a negative reaction from the health workers as they risked being scolded and sometimes humiliated in front of others (28% of relevant codes). This perceived importance, but potential negative consequences, of reporting are reflected in the quotes below:

“[ANC staff] are the ones who will know the medicines to give. So whether you are scolded or not you have to tell them because, when you [are] scolded and given drugs to cure yourself, you are better off" [Older community participant, Ghana]

“Some nurses don’t know how to handle patients so you just fear that she may scold you in front of people. So you just keep quiet even if you have a problem” [Younger community participant, Kenya]

“[You have to report] the drugs you took before starting antenatal [visits] as, if you have a drug that is not good, she would tell you next time you are pregnant don't take it again.

[Later]: sometimes when you say it the nurses may not be patient with you, so you are afraid that when you tell her she will be annoyed with you” [Older enrolled participant, Ghana]

Therefore, while women said that they should (or did), report everything, they often later said that they would not (or did not). The conversations reflected a general fear of health workers' attitudes, with older women in Ghana also saying that it was easier to converse with older nurses who were more polite. When asked about substances they had used during pregnancy, women across all strata said they particularly feared reporting their use of traditional medicines, to a lesser extent over-the-counter medicines and alcohol. There were numerous descriptions of personal and others' experiences of being scolded for using these, including women being accused of using unreported traditional medicines during labour to account for a difficult birth.

There were exceptions, with sporadic descriptions of good relationships with ANC staff in general, where use of such substances was discussed. Moreover, there was limited mention in Ghana and Uganda of ANC staff endorsing the pregnant women's use of traditional medicines, and of participants' perceptions that nurses accept its benefits:

“I know traditional medicines work better than what the modern health workers tell us ……When you tell them they don’t accept [it], yet they also use [it] and they will never tell you that they [do]” [Younger community participant, Uganda]

Smaller categories in this theme related to memory and the ability to name medicines (12% of relevant codes). There was little explicit mention of these impacting on reporting. In fact, women in community FGDs in all countries and age groups said they do remember everything taken during pregnancy: “*They remember alright but they will not say*” [Older community participant, Ghana]. However, these concepts were sometimes implicit in the transcripts; women talked about substances they had used but couldn't remember or know their names and ingredients, particularly herbs.

Suggestions from women about how they could more readily report at the ANC what medicines or other substances they had used reflected the dominance of fear of reporting as a barrier. Thus they recommended that nurses be more understanding/empathetic. In Uganda, where this was discussed at length, enrolled women explained how they were able to forge a special relationship with the friendlier, dedicated registry nurses who sought, and would accept, full disclosure. Similarly, when the registry rationale and method was explained to them, community women considered that being enrolled would help them report such information. There was, however, some indication that this improved attitude of registry nurses did not always extend to those women not enrolled:

- Participant: You don’t talk about [Waragi, a local gin] here because you may find a health worker who may be harsh on you why you took [it].
- Facilitator: What about here in the registry, would you still not talk about Waragi?
- Participant: Here I know I have my nurse who works upon me, so … if she asks me I can let her know that I take a little Waragi, not to be [a] drunkard.
- Facilitator: What of the local herbs?
- Participant: I also have to tell her.
- Facilitator: Won’t she shout at you?
- Participant: No, she doesn’t shout at me

[Younger registry participant, Uganda]

“[Registry nurses] welcome you and give you a lot of care and even ask how you are feeling. But if another person who is not [enrolled in the registry] comes they just look at her like this, yet they are the same nurses who welcomed you well” [Older registry participant, country anonymised]

The registry also facilitated information sharing by encouraging enrolled women to collect names or examples (e.g. leaves, packets) of what they had used, with community groups also acknowledging this could be possible: "*With respect to the herbal medicine, when you can’t remember the name, you will come back and pluck it for the doctor, that this [is] the drug I have taken*" [Older community participant, Ghana]. In addition, women said that making antenatal care more accessible, particularly to those in rural areas, would be beneficial for reporting:

"I also support the home visit because the atmosphere will be conducive to reveal all your health matters, as you are relaxed. Rather than the hospital where you are in a hurry to clear the line for next patient and allow the busy nurse [to] continue with her ever busy schedule" [Older registry participant, Kenya]

### Influences on formal antenatal care

The analysis showed two categories related to formal antenatal care which impact on medicines used in pregnancy and the recall period for reporting; good or poor use of the ANC. The transcripts revealed that good use relied on women seeing the value of going to the ANC if, for instance, it was considered better than alternatives (e.g. traditional care givers). This was apparent in all strata and was particularly important to women who had experienced a previous poor pregnancy outcome while relying on other care options. Some women had practical (including financial) or moral support from family or friends to attend. However, while there was limited evidence of pregnancy testing and ultrasounds being obtained at private facilities early in pregnancy in all countries, it was common in all strata to delay attendance at the government facility until the second or third trimester. Many women said they delayed attending the ANC until they experienced illness, while others intended to go only once to collect an ANC card/book. This was essential in case they needed to use the labour ward, as they anticipated abuse for not registering if there were problems during delivery. Poor attendance was also due to negative perceptions of the ANC (an expectation of being ill-treated by staff, sitting for a long time in a queue or having to undergo an HIV test that they did not want). The narratives showed that younger women were particularly vulnerable to inadequate formal ANC care. They were more likely to have not recognised the pregnancy signs and symptoms, have had less access to support, may have hidden the pregnancy initially, or attempted abortion.

Traditional birth attendants were consulted in all three countries by both those who did and did not attend the ANC regularly, particularly for positioning the baby in the womb. Other options for healthcare during pregnancy included drug shops, traditional healers or herbalists (though these were all less common as they were not considered pregnancy experts), family or community members, and spiritualists. Traditional medicines were widely used across all strata (58% of codes relating to substance-use in pregnancy). Ingested traditional medicines, including leaves and roots sometimes mixed in clay or soil, were taken for fever/malaria, constipation, heartburn, to treat or provoke vomiting (the latter to clean dirt or illness from the mother or baby), anaemia, to prepare for birth (by cleaning the womb of sexually transmitted diseases or for softening the pelvic bones and position the baby correctly), to prevent miscarriage, for the mother's energy, for growth/strength of the baby, or as an abortificant. Some of these medicines were named (typically those herbs picked by oneself or a relative), while others were not (typically concoctions prescribed by a healer). There was a particular discussion among older women in Ghana about not liking medicines from the ANC (such as iron and antimalarials), with one woman saying that, even if she attended the ANC to prevent conflict or condemnation, she did not trust such medicines:

“I will go to the health facility alright. If I don’t go you will tell me [off]. So I will go and when I am given the drugs I will use them as pillows (put them aside). It’s the traditional ones that I take, and [I] am strong too” [Older community participant, Ghana]

Participants in various FGDs reported using the following allopathic medicines during their pregnancies: ibuprofen, aspirin, indomethacin, antimalarials, penicillin and amoxicillin. Some of these were inadvertently taken before the pregnancy diagnosis. For antimalarials this early exposure could reflect conflated narratives about fever, malaria and pregnancy signs/symptoms apparent in all strata:

“I used to miss my periods for even three months, so when it happened I thought it was just malaria. I bought some tablets then came to hospital. It is then that they confirmed that I was pregnant” [Younger community participant, Kenya]

Women rarely mentioned traditional medicines as the cause of poor birth outcomes apart from those specifically used for attempted abortions. Poor outcomes were, however, said to be predominantly due to spiritual attacks (20% of relevant codes), but also the use of over-the-counter drugs (5%), prescribed contraceptives (10%) or being passed directly from parent to child (16%). There was confusion about alcohol throughout all strata. Some women felt that drinking it could harm the mother or baby, but there was evidence of others endorsing it, generally in small quantities, to clean the womb or baby of semen, infection or ingested substances (such as clay), and for ensuring the baby had nice whites of the eyes. Its use was even occasionally sanctioned by ANC staff. Alcohol was also used for enhancing women’s appetites or sleep, and for easing labour:

“People say that Akpeteshie (a local gin), is not good. Well I confess I drink Akpeteshie when I am pregnant because they say that, if you are pregnant and you drink it, your baby will be clean. If you are pregnant and you drink alcohol, you do not get drunk, no matter the quantity you drink” [Younger community participant, Ghana]

- Participant: You should take Guinness (a strong beer)
- Facilitator: Why Guinness?
- Participant: They say when you take it during delivery it cleans the womb, so at delivery there is no stomach ache, the womb is washed clean.
- Facilitator: How much do you take?
- Participant: You take a glass. You take towards delivery, or even during pregnancy, but just once in a while you take the small Guinness.
- Facilitator: Who says this?
- Participant: Just people. But not the doctors of course, but women who have delivered previously and have actually used it to avoid the pain at delivery

[Older registry participant, older, Kenya]

### Social context of pregnancy healthcare-related behaviour

We observed two categories relating to societal influences on women's healthcare-related behaviour during pregnancy; an apparent pressure to conform to what was expected by the ANC, the family and community, and responsibility for a healthy baby. Underpinning the themes reported above was an explicit or implicit understanding that women were expected by the government ANC to attend ANC regularly, to largely abstain from self-medicating, and to report their health and treatment use accurately. Meanwhile, women in all strata showed how they may also have to adhere to certain culturally-dictated behaviours to ensure a healthy pregnancy, and manage the pregnancy at home. The latter was compounded by problems accessing formal care. Expectations of, or advice from, the different parties involved were often conflicting, and involved power relationships. For instance, the conversations with younger women revealed that they were sometimes pressurised by older women in the community to behave a certain way or disregard institutional recommendations for formal care. Over and above their own desires for a healthy baby, conversations also reflected women's responsibilities for producing babies acceptable to the father, his family, and/or the community. All FGDs showed how women must be careful about how they conduct themselves in public, what they eat or what medicines they use, as these may dictate whether a baby is born healthy. Despite these considerations, she may still be blamed for producing a child with a birth defect. These societal pressure categories are demonstrated below:

“The old women … advised me that it is not necessary to go early [to the ANC] because you will be going there all the time. I told them that I want to go to the health centre because I was feeling lower abdominal pain. Still they rejected me and could tell my husband not to give me money for going there because my pregnancy was still young. So I just came when he gave me money to eat” [Younger registry participant, Uganda. Attended ANC first at 5 months]

"In my hometown, they say pregnant women should not engage in a lot of things, so if you turn a deaf ear to what the tradition says and there [are] gods in your town, [you] could have a child with a defect. Therefore if you live in such a town you should be very careful until you deliver" [Older community participant, Ghana]

"I also have a sister-in-law who gave birth to a child with a big head but the husband chased her and told her that he doesn’t produce babies with big heads" [Older registry participant, Uganda]

"When one is pregnant you don’t have to take alcoholic drink because it affects the babies in the womb and when you give birth the babies will not look attractive and people would not give you a helping hand in carrying him or her" [Community participant, older, Ghana]

## Discussion

This qualitative study found that women desired, and felt responsible for, carrying a healthy baby to term. However, despite largely deeming all health-related information during pregnancy important to report at the ANC to help ensure a good outcome, they could not always recall or give exact details of what substances they had used. Moreover, the pressure to conform to both formal and traditional pregnancy care options, or a practical need to combine such care, led to a common use of substances with unknown effects in pregnancy, including traditional medicines, over-the-counter medicines and alcohol. Such substances were not always reported easily at the ANC as women generally feared being admonished by staff. While pregnancy registries could play a valuable role in building knowledge about the safety of medicines used in pregnancy in resource-limited areas, they are dependent on valid and reliable data. If there is significant measurement error of exposures, outcomes or confounders, associations will be incorrect and pregnant women will not get optimal advice about suitable medicines. It is, therefore, important to consider all these barriers to accurate participant reports so that socially-contextualised solutions may be sought.

This present finding that various factors influence women’s willingness or ability to seek antenatal care is consistent with results of previous studies [18-20]. As well as increasing the risk for complications, those not adhering to national recommendations for such care may be less likely to receive appropriate advice about safe substance use. This will be exacerbated should women not recognise, or choose to hide their pregnancy, or lack family or community support to seek appropriate healthcare, particularly in younger women [21,22]. However some women indicated that the advice they receive about substance use in pregnancy could be inconsistent; midwives sometimes endorsed the traditional medicines and alcohol that women largely expected to be vilified for reporting. This confusing situation for pregnant women probably reflects the staff's own dilemmas with interpreting mixed messages when living and working in a context where traditional medicine is an embedded practice and sometimes the only readily-available option.

The registry fostered a relationship between pregnant women and ANC staff where some women said they were more able to report everything they had used. Indeed, preliminary data from the registry database shows that use of herbal medicines and alcohol were reported in all countries. However, this qualitative study cannot confirm whether the extent of such reporting is higher than in non-registry settings or reflects the true experience of enrolled women. If the registry did enhance reporting, this may have been influenced by other factors. For instance, the informed consent process explained why it was important to report everything, and the data collection tool required ANC staff to explicitly ask participants about their use of traditional and herbal medicines and alcohol, with space to record these details. The latter is in contrast to most standard ANC records or booklets in these countries.

There was an indication that an improved relationship between patients and staff may not extend to other women attending the ANC who were not enrolled in the registry. Enrolling all women at a particular facility in a drug exposure surveillance system would ensure that the community does not become resentful of the project or those included. However, whether it is feasible for any woman presenting at a busy ANC to receive the same attention, and feel free to report her experiences accurately, needs further investigation. As a minimum, registry sites should support the various interventions to encourage respectful maternity care, including improved working conditions for ANC staff [23]. Staff should also be guided in how to collect data on substances whose use they are typically trained to discourage. Emphasis should be placed on conveying messages about known risks or the fact that there are limited safety data for some substances, so that pregnant women understand the rationale for discouraging their use [24-26]. Much could be learnt from research on smoking in pregnancy, to encourage transparency and trust in ANC communications about potentially risky behaviour [27].

Poor ANC attendance will undoubtedly impact negatively on registry participants remembering their health and treatment experiences accurately. There is a dearth of research relating to the validity of pregnant women's recall of non-prescription drugs in general, and very little about self-reported medical histories and treatment use in resource-limited settings. However, it is clear that some questioning techniques are better than others [28]. For instance, structured questions indicating specific health conditions and names of treatments are likely to increase the sensitivity of their detection compared with open questions [10,14]. As detailed questionnaires place a burden on staff, the limited questions incorporated into the WHO registry pilot study reflected its intention to be adopted by busy ANCs. This may have led to under-reporting of drug exposures and developing an optimal questioning method in these contexts requires further work. Antenatal records could also be designed to encourage elicitation and recording of drug exposures by staff as a routine practice. Pictures of typical health complaints and medicines may be needed in areas with low literacy, and registry teams could learn from other pharmacoepidemiologic interview techniques, whereby participants are helped to identify the timing and duration of medicine use [29]. These may be particularly useful in areas where women present late in pregnancy for their first ANC visit.

Women enrolled in the registry were happy to keep records or collect samples of what they had taken. However, the constituents and doses of herbal remedies, such as concoctions dispensed by traditional practitioners, are often unknown by consumers [5]. It is theoretically possible that this information may even deliberately be withheld for fear that consumers may then source their own herbs. This problem of not knowing ingredients of such substances is supported by preliminary registry findings where these details were not always obtained. It may therefore only be feasible to capture a binary yes/no for herbal use in registries, to identify signals of potential harm for later, more specific, studies. However, certain concoctions are well known locally to be used in pregnancy and may represent a semi-standardised regimen in some regions. If these were adequately recorded at the ANC it may be possible to establish any associated risk of harm. For allopathic medicines, registry sites could pursue intra- and inter-institutional record linkages (with other clinics, outpatient departments, labour and neonatal wards) and opportunities for innovative collaborations with other local healthcare providers, including local private clinics and traditional birth attendants. There may be scope for participant referrals or data sharing, and exploring local understanding of substances that are typically used or prescribed.

This study was concerned with the complex field of obtaining accurate reports from registry participants about their exposures to medicines and other substances. Finding appropriate questioning methods in pharmacoepidemiological studies is methodologically challenging. For instance, these participants voiced their fears of mentioning traditional medicines, and yet they also considered them as having less potential for causing poor pregnancy outcomes compared to other perceived determinants, such as spiritual attacks. The latter may reflect a global public perspective that complementary medicines are generally safe [30]. Judgements made by participants about the significance of a health event or treatment, or its relevance to the situation, can impact on reporting [10,13], and substances considered by consumers as safe may potentially be deemed irrelevant to report. Due to the lack of a gold standard for participant reported data in pharmacepidemiology, it is therefore important that qualitative methods be incorporated into any future work, to contribute to understanding the nuances involved.

### Limitations

We could not verify that the reports of women taking part in these FGDs are accurate. Although the questions in the FGD guide were applied in all FGDs, there were differences in how facilitators probed the answers. Therefore some FGDs generated richer data which may impact on the findings. Our results may not be generalizable to other studies where the methods and context are very different. Ghana was unable to achieve 2 FGDs in the registry participants' strata, and there was an error during recruitment whereby some women over the age of 51 were enrolled into some of the 4 community FGDs. As the Ghanaian FGDs had the richest data it is likely these issues did not compromise the comparisons between sites.

## Conclusion

There was widespread reported use of traditional medicines in pregnancy in this study's population, and, to a lesser extent, use of over-the-counter medicines and alcohol. The qualitative findings suggest that the WHO registry methods could address some barriers to reporting these at the ANC as it gave women tools to help overcome problems with recall and naming of substances, and sometimes enhanced their relationship with ANC staff. Questioning techniques for women attending busy ANCs where registries are implemented should be explored to identify optimal approaches, especially for recording substances such as herbal concoctions where the detail of constituents may never be known.
